# Experimental Studies of the Machinability of SiCp/Al with Different Volume Fractions under Ultrasonic-Assisted Grinding

**DOI:** 10.3390/ma17123024

**Published:** 2024-06-20

**Authors:** Chen Hu, Yongwei Zhu, Ruoxun Fan

**Affiliations:** 1 College of Mechanical Engineering, Yangzhou University, Yangzhou 225127, China; huchen198804@163.com; 2 College of Transportation Engineering, Yangzhou Polytechnic Institute, Yangzhou 225127, China; fanruoxun@126.com; 3 JITRI Institute of Precision Manufacturing, Nanjing 211800, China

**Keywords:** SiCp/Al, ultrasonic-assisted, volume fraction, grinding machining

## Abstract

High-volume fraction silicon carbide particle-reinforced aluminum (SiCp/Al) has a promising application for its high specific strength, wear resistance, and thermal conductivity. However, SiCp/Al components with a high-volume fraction are prone to poor surface quality and defects such as fractures, cracks, and micro-pits. It has been reported that ultrasonic-assisted grinding machining (UAG) helps to improve the quality of SiCp/Al machined surfaces. However, the differences between SiCp/Al with different volume fractions obtained by UAG machining are not clear. Therefore, a comparative study of surface roughness, morphology, and cutting force was carried out by UAG machining on SiCp/Al samples with volume fractions of 45% and 60%. Compared to the 45% volume fraction SiCp/Al, the 60% volume fraction SiCp/Al has a higher cutting force and roughness under the same machining parameters. In addition, experiments have shown that cutting forces and surface roughness can be reduced by increasing the tool speed or decreasing the feed rate. UAG machining with an ultrasonic amplitude within 4 μm can also reduce cutting forces and surface roughness. However, more than 6 μm ultrasonic amplitude may lead to an increase in roughness. This study contributes to reasonable parameter settings in ultrasonically-assisted grinding of SiCp/Al with different volume fractions.

## 1. Introduction

The application of SiCp/Al material has rapidly improved, because of its advantages of high specific strength, good wear resistance, and a low coefficient of thermal expansion [1,2]. However, the hardness of SiCp/Al makes it prone to excessive cutting forces [3], severe tool wear, poor surface quality, and even defects such as fractures, cracks, and micro-pits [4,5,6]. These defects will reduce the expected life of the product or cause it to scrape because of their negative impact on fatigue behavior. This hinders the wide application of SiCp/Al composites; hence, some scholars have investigated the machining of SiCp/Al composites using novel machining [7,8,9]. In recent years, UAG machining has developed rapidly, and it has good prospects for application. UAG offers significant advantages in machining high-hardness materials because it compounds the material removal mechanisms of ultrasonic machining and conventional grinding (CG) machining. It is reported that surface roughness can be reduced and machining quality can be improved, while maintaining efficient material removal [10,11,12,13,14].

Ding et al. conducted an experimental study with UAG machining. Its results revealed that the cutting force by UAG machining was significantly lower than that by CG machining, and its surface roughness and contour fluctuation height were reduced [15]. Denkena et al. investigated the roughness and cutting forces after UAG machining and analyzed the effect of machining factors such as depth of cut, feed rate, and ultrasonic amplitude. They pointed out that the effect of ultrasonics will reduce the influence of tool microgeometry on the surface roughness after machining [16]. Liu et al. established a cutting force model for UAG machining based on the brittle fracture theory and analyzed the influence of each factor on the cutting force through experimentation [17].

The above studies have deepened our understanding of the processing mechanism of UAG. In terms of material applications for UAG machining, most of the early literature investigated SiCp/Al with a low volume fraction (10~35%). In recent years, with the increase of high-volume fraction (45~75%) SiCp/Al applications in aerospace and drones, research on the machining application of high-volume fractions has become a new focus. Cutting forces during machining [18,19], surface morphology after machining [20], and roughness [21] are the most prominent issues in these discussions. Based on the brittle fracture theory, Zhou et al. established a UAG machining model, which was verified by experiments on SiCp/Al material with a 45% volume fraction [21]. In addition, the machined surface quality, tool wear, and chip shape were studied. It is reported that the surface roughness could be reduced by an average of 11.53% by UAG machining [22]. Zheng et al. investigated the three-dimensional surface roughness and fractal dimension of SiCp/Al machined by UAG. Orthogonal experiments showed that the main factor affecting the surface roughness was the tool speed, followed by the ultrasonic amplitude, depth of cut, and feed rate [23]. Wang et al. developed a microstructure-based finite element model in which the properties of SiC particles, the Al matrix, and the interfacial layer between SiC particles and the Al matrix are considered. In addition, an experiment with a 45% volume fraction of SiCp/Al was used to analyze the deformation and failure behavior of composites in the ultrasonic vibration-assisted grinding process [24]. Gu et al. established a prediction model for the surface roughness of ultrasonic vibration-assisted grinding of SiCp/Al composites. Compared with the experimental results, its accuracy was verified [25].

High volume fraction SiCp/Al materials are characterized by high hardness and a high modulus of elasticity, which makes them suitable for components with requirements for impact, fatigue, or wear resistance. Low volume fraction SiCp/Al materials, on the other hand, offer higher thermal conductivity and a lower density, making them ideal for components that require heat dissipation. The mechanical properties of SiCp/Al materials with different volume fractions, such as hardness and modulus of elasticity, differ considerably. Therefore, it is necessary to investigate their processing differences under UAG machining. Veličković et al. analyzed the impact of adding a small percentage of silicon carbide (SiC) particles into an aluminum matrix with nanocomposites of 0, 0.2, 0.3, and 0.5 wt.% SiC. They noticed that a small amount of SiC particle reinforcement (0.2 and 0.3 wt.%) does not improve the wear resistance of the nanocomposite; however, nanocomposites containing 0.5 wt.% of the reinforcement have a higher tensile strength and wear resistance [26,27]. Xiang et al. performed scratch experiments with a single diamond particle on materials with volume fractions of 25%, 40%, 55%, and 70%. These experiments showed that the width of the scratch profile increased significantly with an increase in volume fraction [28]. Therefore, the volume fraction has an important influence on the machining effect. 

Most of the current literature has only investigated the UAG machining of single volume fraction SiCp/Al materials; therefore, this the objective of this research is to innovatively examine the differences in the machinability of SiCp/Al materials with different volume fractions under UAG machining and its mechanism. Several groups of one-factor tests were carried out by varying different processing parameters. The machinability of SiCp/Al materials with different volume fractions was investigated in terms of surface morphology, roughness, and cutting force. The influence and mechanism of the factor of volume fraction during UAG machining were also analyzed. This paper is divided into three main sections. The first is the materials and methods section. This section describes the material properties, the experiment setup, the experiment procedure, and the measurement device. Next is the experiment results and analysis section. The section discusses and analyzes the effects and mechanisms of various machining parameters on surface roughness, cutting force, and morphology. Finally, there is a conclusion and summary section.

## 2. Materials and Methods

### 2.1. Material Properties

**Figure 1:**
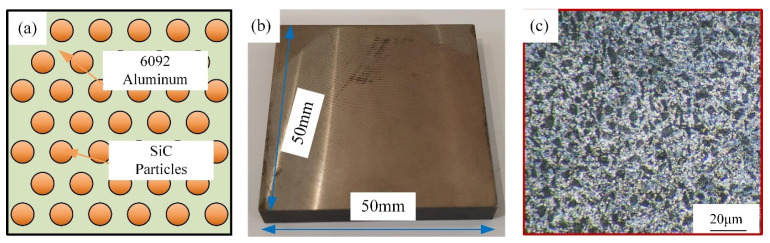
(**a**) Material schematic; (**b**) Sample appearance; (**c**) Microscopic morphology.

As shown in the schematic diagram (Figure 1a), the SiCp/Al sample used for machining was composed of the matrix (6092 aluminum) and the reinforcing phase (SiCp particles). Volume fractions of 45% (Sample A) and 60% (Sample B) were used as machining materials for the experiments to investigate the machining differences between them. The samples were heat-treated in T6 conditions to improve their hardness and strength. The mechanical properties of the two samples are shown in Table 1. 

As shown in Figure 1b, the size of the sample was 50 mm × 50 mm × 5 mm. Figure 1c shows the microscopic morphology of Sample A after grinding and polishing. It can be seen that the SiCp particles are uniformly distributed in the aluminum matrix with a diameter of about 5 μm.

### 2.2. Experimental Equipment

**Figure 2:**
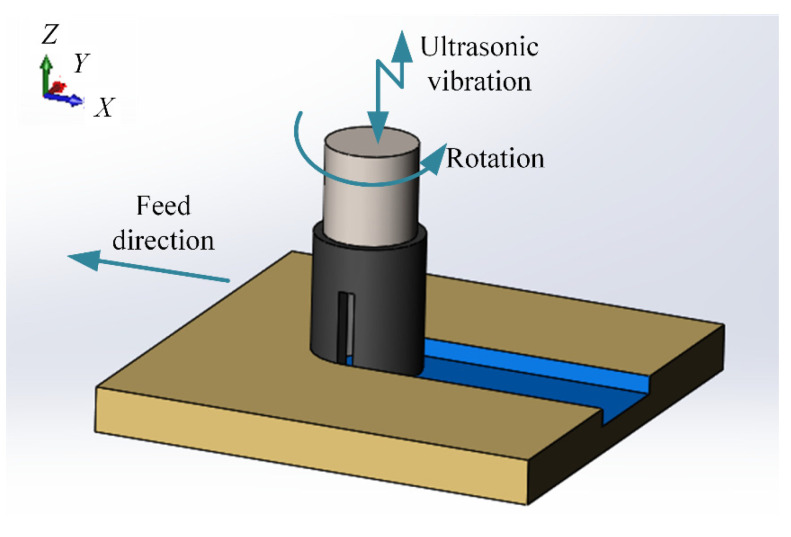
Schematic diagram of UAG processing.

The UAG machining mechanism is shown in Figure 2. During the machining process, the motion of the grinding tool can be regarded as a mixture of three modes of motion: the first is the feeding motion in the X direction; the second is the rotary motion with the tool axis; and the third is the ultrasonic vibration along the Z direction. The workpiece was cut continuously under the compound motion of the grinding tool, thus achieving high efficiency in the removal of the material.

**Figure 3:**
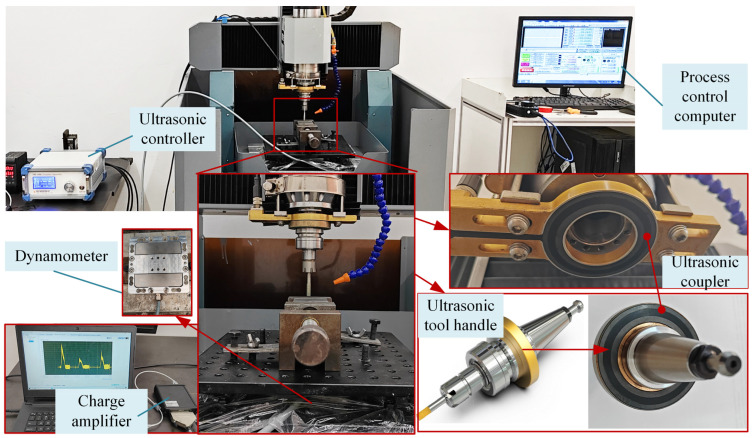
Experimental equipment.

As shown in Figure 3, UAG machining consists of a conventional grinding machine (DS4040) and an ultrasonic machining machine, including an ultrasonic toolholder and ultrasonic controller. The ultrasonic toolholder holds the grinding tool securely with an ER16 collet and enables the tool to vibrate ultrasonically in the Z direction simultaneously with the rotary motion. The ultrasonic controller has an on/off switch and an amplitude adjustment knob. The switch enables switching between CG and UAG processing. The adjustment knob enables the ultrasonic amplitude to be adjusted in the 8 μm range. The ultrasonic toolholder is indirectly powered by an ultrasonic coupler. The coupler enables wireless transmission of the ultrasonic power supply, allowing the machine to maintain a stable power supply at maximum speeds of 25,000 r/min.

In order to measure the cutting forces during machining, the workpiece clamping vise is not mounted directly on the machine tool but is fixed to an adapter stage equipped with a dynamometer. This ensures that the cutting force can be measured in the X, Y, and Z directions in real time during machining.

**Figure 4:**
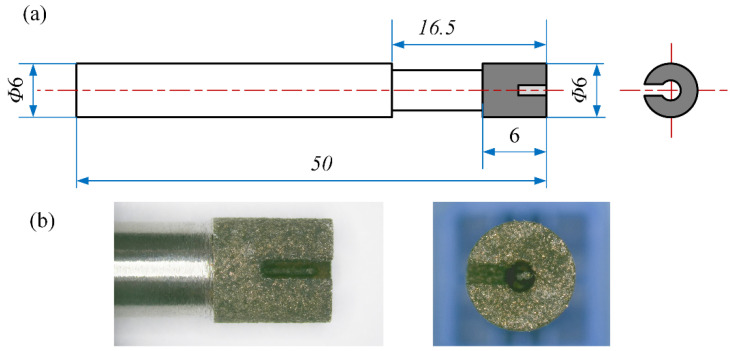
Grinding tool: (**a**) Schematic diagram of tool structure; (**b**) Morphology of grinding head.

Since SiCp/Al is a high-hardness material, a metal-sintered diamond grinding tool, as shown in Figure 4, was used as the machining tool for the experiment. The tool shank and grinding head are both 6 mm in diameter. The grinding head has a hollow and a groove so that the chip can be easily discharged during machining. The abrasive grains of the grinding head are made of SDC diamond with a diameter of about 150 μm, which are uniformly distributed in the metal matrix.

### 2.3. Experimental Procedure

Since ultrasonic amplitude (*A_z_*), feed rate (*v_w_*), tool speed (*v_s_*), and depth of cut (*a_p_*) are critical parameters in machining, these four machining parameters were chosen as factors in the experiment. Due to the power limitation of the ultrasonic equipment, the maximum value of *A_z_* is 8 μm, so 0–8 μm was selected as the parameter variable. Since the maximum rotational speed at which the UAG machining system can ensure a smooth experiment is 25 krpm, parameters within the range of 0–21 krpm were selected as parameter variables for *v_s_*. The variables *v_w_* and *a_p_* were selected as references [29]. The reliability and stability of machining will be affected if these values are set too large.

Twelve groups of experiments were set up to investigate the machining characteristics of SiCp/Al materials with different volume fractions. The specific conditions of the experiments are shown in Table 2. To maintain comparability, each group of experiments was machined along the X direction of the machine, as shown in Figure 2. Different machining parameters of the same group were continuously machined at the same Y position. Different groups were processed in different Y-direction positions to maintain the independence of each group. In order to ensure accuracy, the experiments were repeated three times for each group, and the average value was taken as the result for analysis. 

### 2.4. Measuring Device

The factors to be measured in this experiment included ultrasonic amplitude, machining cutting force, surface roughness, and surface morphology. The ultrasonic amplitude was measured by the LK-G5000 (KEYENCE, Osaka, Japan) series laser displacement transducer in conjunction with the LK-H020 sensor head, with a maximum sampling frequency of 100 kHz and a repeatability of 0.02 µm. 

The surface roughness was measured by a roughness measuring instrument (model TR200, DANA, Huzhou, China), which can accurately measure the surface measurement values. The arithmetic mean deviation (Ra) of the contour of the surface was measured to characterize the roughness of the surface.

**Figure 5:**
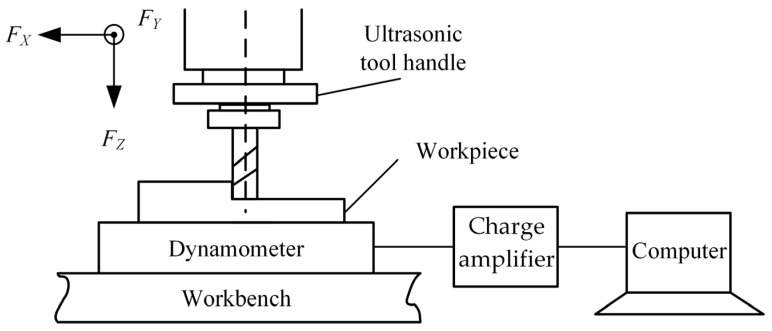
Principle of cutting force measurement.

As shown in Figure 5, the cutting force measurement system consisted of three parts: a dynamometer, a charge amplifier, and a computer. The cutting force was measured by a dynamometer (model ME-K3D120, ME-Meßsysteme, Hennigsdorf, Germany). This converts the force signal into an electrical signal, which is amplified by a charge amplifier (model GSV-4USB, ME-Meßsysteme, Hennigsdorf, Germany) and transferred to the computer via the USB interface. The cutting force can be displayed and read out via the accompanying software.

Due to the feeding of the tool along the X-direction and ultrasonic vibration along the Z-direction, the cutting force is mainly reflected in the X-direction and Z-direction. In order to comprehensively evaluate the cutting forces, the forces in the three directions were synthesized into a combined cutting force *F* for comparison, which was calculated as follows:

(1) $$F=\sqrt{F_{X}^{2}+F_{Y}^{2}+F_{Z}^{2}}$$

where: *F_X_*, *F_Y_*, and *F_Z_* are the average cutting forces measured by the force measurement system in the X, Y, and Z directions, respectively, during stable machining.

The surface morphology was observed by a scanning electron microscope (SEM, model Gemini SEM 300, Zeiss, Jena, Germany), which can realize magnifications up to 2 million times. In addition, this was equipped with energy dispersive X-ray spectroscopy (EDS) for qualitative and semi-quantitative analysis of samples, which provides a comprehensive understanding of the relationship between sample composition, microstructure, and properties. The morphology of inlet edges was observed by a 3D digital microscope (model ALSVI 3DM-HD228S, Shenzhen, China), which makes it easier to adjust the observing angle.

## 3. Results and Analysis

### 3.1. Surface Morphology

**Figure 6:**
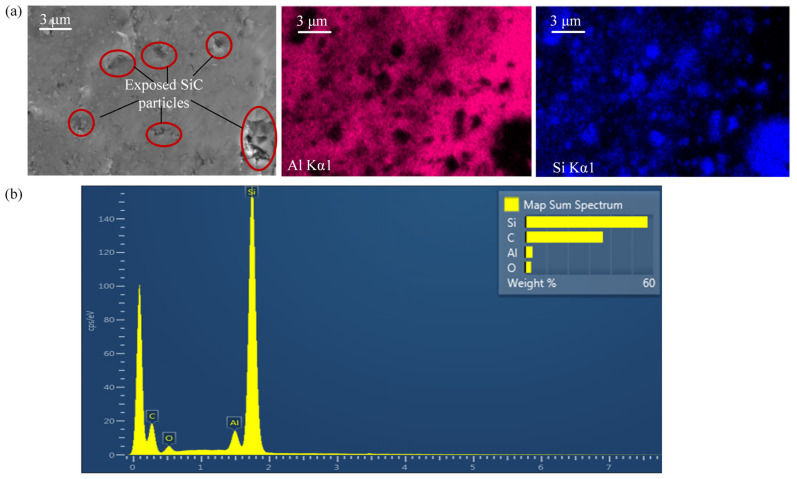
(**a**) EDS analysis of sample A after UAG machining with 4 μm. (**b**) Distribution of elements.

Since the processed aluminum matrix and SiCp particles are irregularly mixed together, it is difficult to distinguish them by SEM observation. Therefore, energy dispersive spectroscope (EDS) analysis was used to realize the differentiation between SiCp particles on the surface and the aluminum matrix [30]. The EDS surface sweep analysis of sample A after UAG machining with 4 μm is shown in Figure 6a. It can be seen from the distribution of Si and Al elements that the main component of these particles is SiCp. Only a few large SiCp particles remain after machining, and most of the particles have been fractured to 1 μm and dispersed in the aluminum matrix. Elemental analysis of these particles was carried out as shown in Figure 6b. It can be seen that the major elements of these particles are Si and C. This part of the analysis provides the basis for the study of the SEM of the machined surfaces.

**Figure 7:**
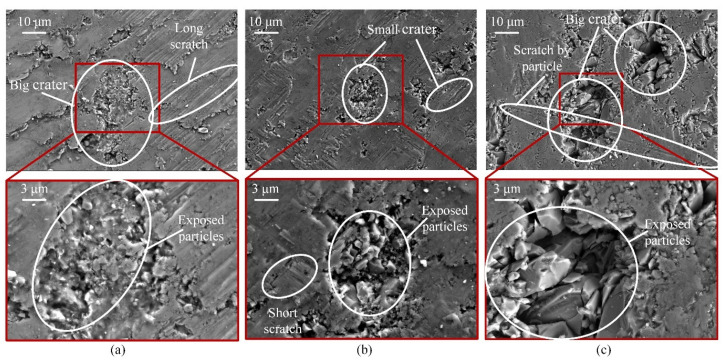
SEM morphology: (**a**) surface of sample A by CG machining; (**b**) surface of sample A by UAG machining with 4 μm amplitude; (**c**) surface of sample B by UAG machining with 4 μm amplitude.

Figure 7a shows the SEM of the CG-machined sample A. It can be seen that the aluminum matrix has long and continuous scratches under the rotating and feeding action of the abrasive grains. Craters with exposed SiCp particles are present in the aluminum matrix. These craters are large but shallow. They are mainly produced by the excision or shattering action under the rotational action of diamond abrasive grains. Due to the weak abrasive coverage of the aluminum matrix during CG machining, these craters are not well covered.

**Figure 8:**
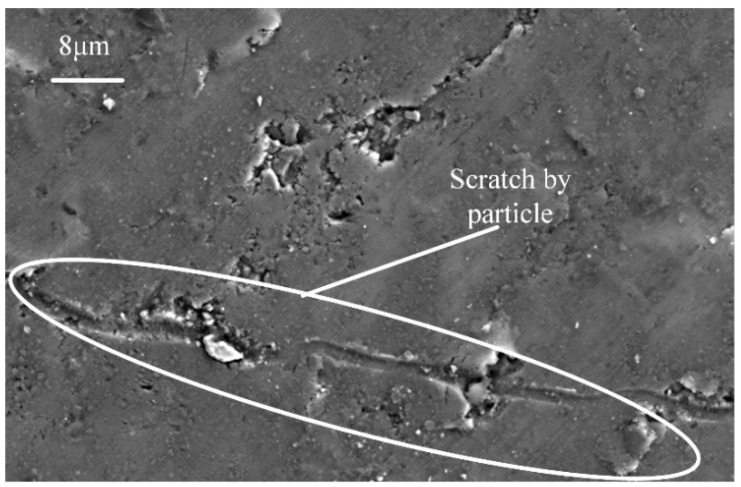
SEM morphology of sample B machined by UAG with 8 μm amplitude.

As shown in Figure 7b, the morphology of the UAG-machined surface is much smoother than the CG-machined surface. Although there are scratches and craters on the machined surface, these scratches are shorter and shallower than those on the CG-processed surface, while the craters are smaller and deeper. The SiCp particles are fragmented by the “hammering” effect of the ultrasonic vibrations, forming aggregated clusters of small particles. Some of the small particles are dislodged, resulting in deeper pits. However, as shown in Figure 8, when the ultrasonic amplitude *A_z_* increases to 8 μm, some long and deep scratches appear on the machined surface, in which large embedded SiCp particles can be found. 

Figure 7c shows the SEM morphology of sample B machined by UAG. It was found that most of the machined areas have craters, and compared with sample A, the quantity and size of these craters are significantly increased. Since the composition of the aluminum matrix is reduced relative to sample A, the coating effect of the aluminum is significantly weakened, making it difficult for the aluminum matrix to cover these crater regions in most areas. Therefore, more large and uneven particles are exposed in the craters. In addition, scratches by particles on the matrix can be observed clearly on the surface, and compared with sample A, these can only be found when the ultrasonic amplitude reaches 8 μm.

**Figure 9:**
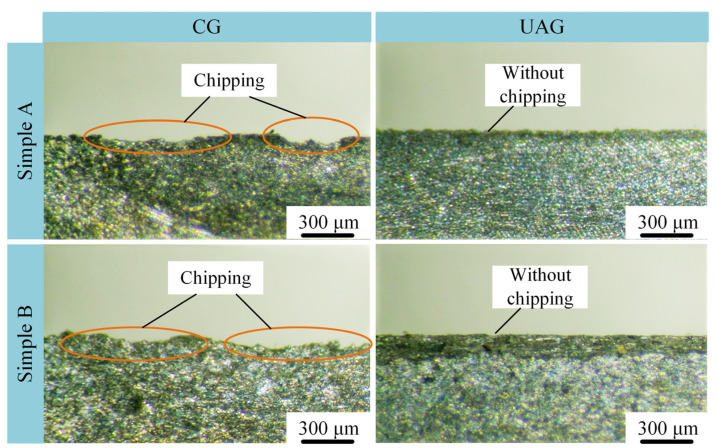
Morphology of the inlet edge of each machining group.

It has been shown in the literature that SiCp/Al materials are prone to defects such as chipping at the edges of the machined inlets [31]. Since the same group is machined at the same Y-direction position and continuously along the X-direction, there is only one inlet edge in the same group. After adjusting the sample at an angle, the inlet edge can be observed with an optical microscope. The comparative results of the two samples machined in both UAG and CG with the same machining parameters are shown in Figure 9. In the case of CG machining, chipping defects can be clearly observed at the edges of both samples. This suggests that there is a material removal mode of brittle fracture during processing due to the presence of SiCp particles.

In addition, for both sample A and sample B, the finish of the inlet edge of UAG machining is obviously higher than that of CG machining, which indicates that ultrasonic assistance can have a good suppression effect on the fracture damage at the inlet edge. In addition, the chipping at the entrance edge of sample A is lower and smaller than that of sample B with CG machining using the same parameters. While using UAG machining, sample A has a flatter entrance edge than sample B. This indicates that the degree of brittle fracture at the inlet edge increases as the volume fraction of SiCp/Al increases, regardless of the processing method used.

### 3.2. Cutting Force

The cutting forces during machining were measured in real time by a three-way dynamometer. In terms of the magnitude of each component force, *F_Z_* is the largest component of the cutting force. *F_X_* is the second-largest component of cutting force, which is mainly generated by the tool feeding along the X direction. However, *F_Y_* is small and almost negligible. In order to comprehensively compare the cutting forces under each parameter, the combined cutting force *F* was calculated by Equation (1). The cutting forces stated below all refer to the combined cutting forces calculated by this formula.

**Figure 10:**
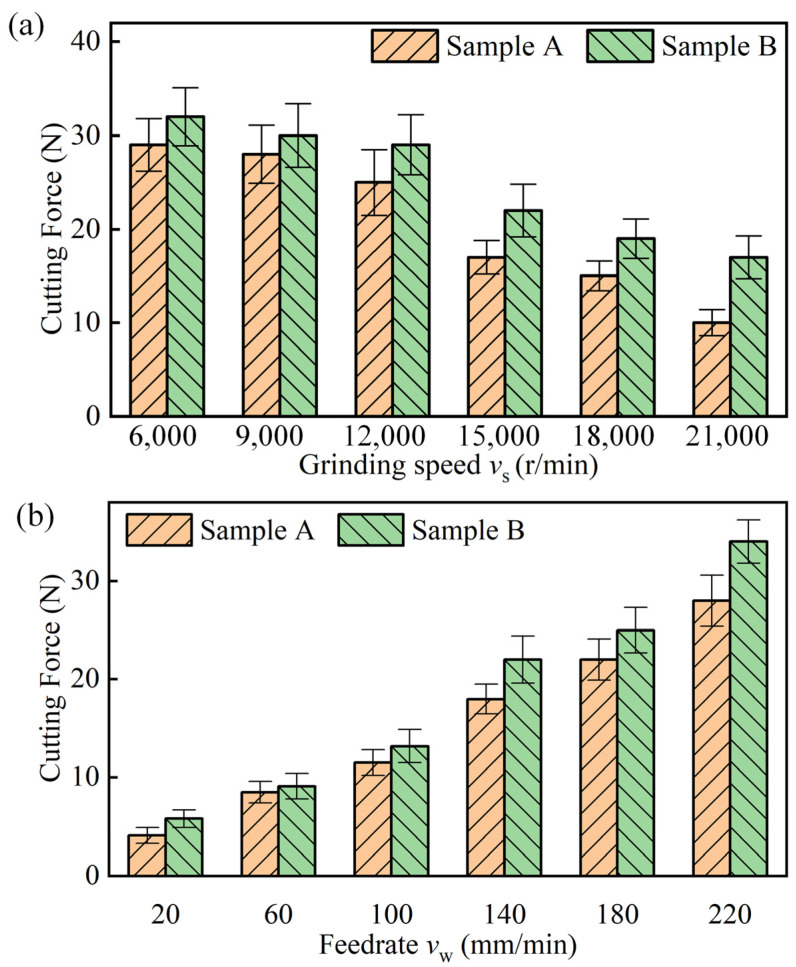
(**a**) Effects of *v_s_* on cutting forces; (**b**) Effects of *v_w_* on cutting forces.

Figure 10a,b demonstrates the effect of *v_s_* and *v_w_* on the cutting force under UAG machining with an *A_z_* of 4 μm. The machining parameter *a_p_* was 0.01 mm. As can be seen from the figure, the cutting force of sample A is always lower than that of sample B, regardless of how *v_w_* or *v_s_* vary. This indicates that the cutting forces required for SiCp crushing are higher than those required for plastic removal of the aluminum alloy matrix.

It can be seen that the cutting force of sample B is always higher than the cutting force of sample A. As the *v_s_* increased from 6000 r/min to 21,000 r/min, the cutting forces of the two samples were gradually reduced. This is mainly due to the fact that, as the grinding speed increases, the frequency of grit cutting increases, resulting in a decrease in the amount of individual grit cutting. The cutting force of sample B was reduced by 46.8%, which is slightly lower than that of sample A (65.5%). For materials with high volume fractions, the reduction in cutting forces due to grinding speed decreases. This is mainly due to the differences in the mechanisms of removal of SiCp-reinforced particles and metal matrix. Since SiCp particles are removed in a brittle form, the cutting force is not reduced to the same extent as in alloys.

Figure 10b shows that the effect of *v_w_* is opposite to *v_s_* for both materials. As *v_w_* increased from 20 mm/min to 220 mm/min, the cutting forces of both samples A and B increased. This is mainly due to the increase in feed rate, which causes an increase in the amount of cutting in a single cycle. In addition, the increased feed rate also leads to a decrease in cooling, which is another reason for the increase in cutting forces. The cutting force of sample A increased up to 6.9 times, while the cutting force of sample B increased slightly less, to about 5.9 times. This suggests that changes in *v_w_* have less effect on materials with higher volume fractions than those with lower volume fractions.

**Figure 11:**
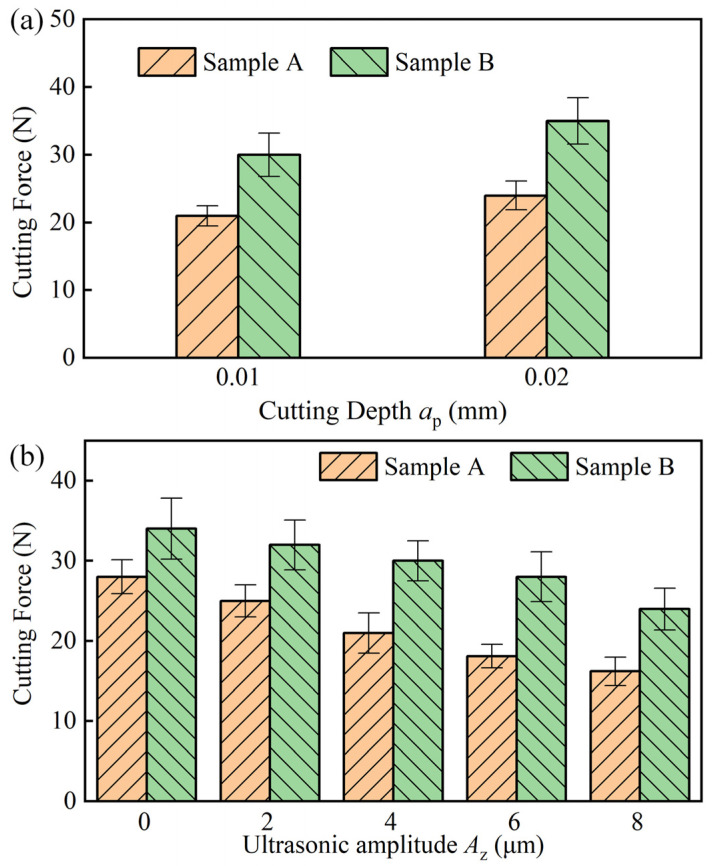
(**a**) Effects of *a_p_* on cutting forces; (**b**) Effects of *A_z_* on cutting forces.

Figure 11a shows the effect of *a_p_* on the cutting forces of the sample parts under UAG machining. The cutting force of sample B is higher than that of sample A for both samples with the same *a_p_* parameters. As *a_p_* increases, the cutting forces on samples A and B increase accordingly. This is mainly due to the fact that the increase in *a_p_* increases the amount of abrasive grain cutting in a single cycle, resulting in greater cutting resistance. When *a_p_* was increased from 0.01 to 0.02, the cutting forces of sample A and sample B increased by 19% and 16%, respectively. The effect of *a_p_* on the material with a volume fraction of 60% is relatively weak compared to the material with a volume fraction of 45%.

Figure 11b shows the effect of *A_z_* on cutting force. It can be seen that, by CG machining (*A_z_* = 0), the cutting forces of the samples A and B reached 28.2 N and 34.1 N, respectively. However, the cutting forces gradually decreased with UAG machining, and the cutting force decreased with the increase in *A_z_*. When *A_z_* was increased to 8 μm, the cutting forces of samples A and B decreased to 16.2 N and 24.7 N, respectively. Compared with CG machining, the cutting forces decreased by 42.5% and 27.6%, respectively. This suggests that ultrasonics can cause a reduction in cutting force for both volume fractions of material, but the impact of ultrasonics is greater for the lower volume fraction material.

### 3.3. Surface Roughness

**Figure 12:**
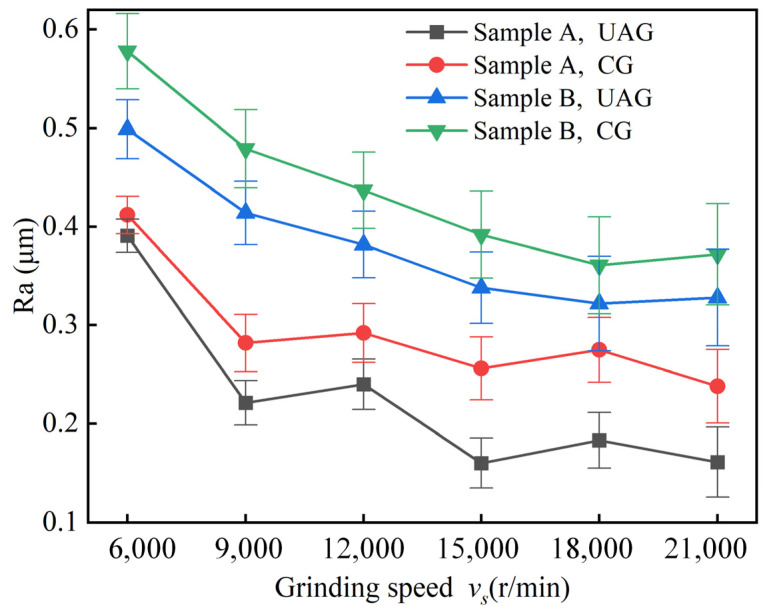
Effects of *v_s_* on Ra.

Surface roughness Ra is an important indicator of machining quality, so the effect of each machining parameter on Ra was analyzed. Figure 12 shows the variation of surface roughness Ra with *v_s_*. The machining parameters *a_p_* and *v_w_* were 0.01 mm and 120 mm/min, respectively. For UAG machining, the parameter *A_z_* was set to 4 μm. When *v_s_* increases from 6000 r/min to 15,000 r/min, the surface roughness decreases significantly with the increase in rotational speed for both samples, whether they underwent CG machining or UAG machining. This is mainly due to the fact that the increase in rotational speed increases the frequency with which individual abrasive grains cut the material, which reduces the cutting thickness of a single cut, resulting in a relatively smooth surface. However, as *v_s_* continues to increase, the roughness Ra no longer decreases; obviously, even an increase in Ra can be found under some of the *v_s_*. The possible reason for this is that, as the *v_s_* rises, the ultra-high rotational speeds cause particle shedding, as shown in Section 3.1, which leads to fluctuations in Ra as the particles scratch the surface.

**Figure 13:**
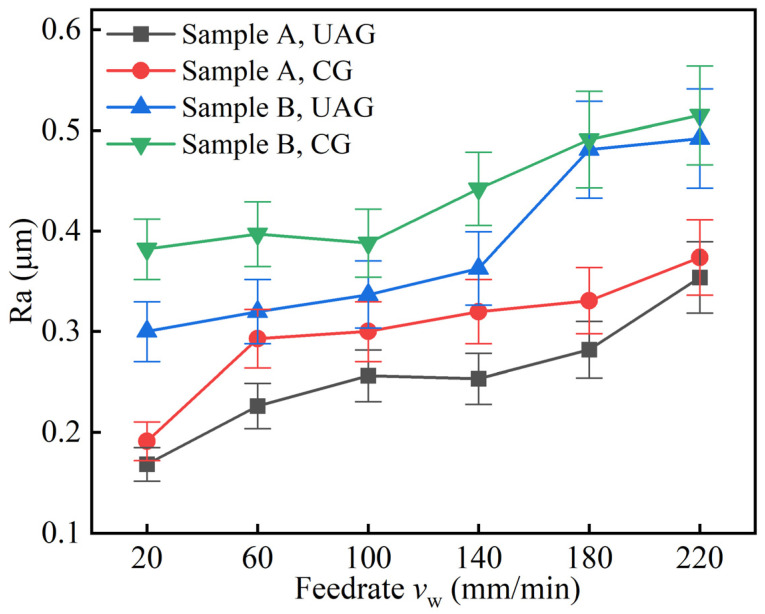
Effects of *v_w_* on Ra.

Figure 13 shows the variation of surface roughness Ra with the machining parameter *v_w_*. The machining parameters *a_p_* and *v_s_* were 0.01 mm and 15,000 r/min, respectively. For UAG machining, the parameter *A_z_* was set to 4 μm. When *v_w_* increased from 20 mm/min to 220 mm/min, the roughness increased gradually, and as *v_w_* exceeded 140 mm/min, the roughness increased more significantly. The reason for the Ra rise can be attributed to two main aspects. One is that, as the feed rate increases, the depth of the crater in a single cut increases, resulting in an uneven surface. On the other hand, the cooling effect gradually decreases as *v_w_* increases. Overheating of the surface leads to deterioration of the machining environment, which in turn negatively affects the surface roughness.

**Figure 14:**
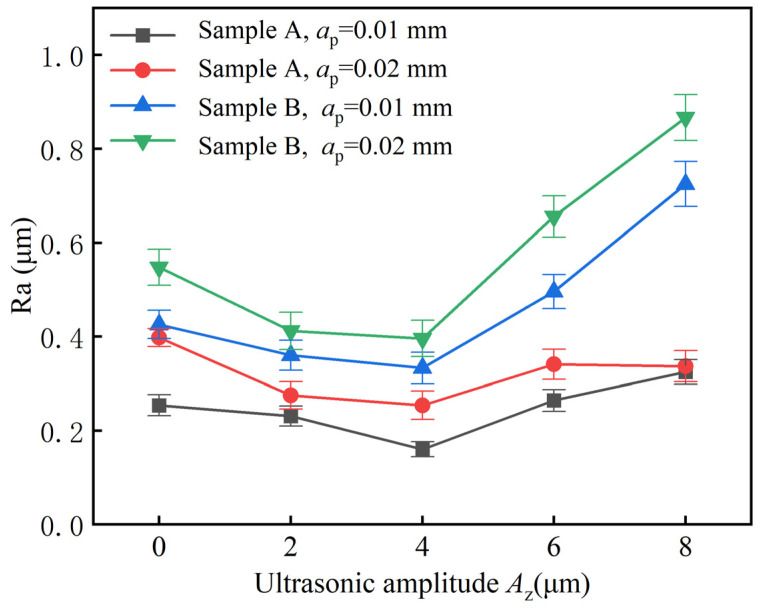
Effects of *A_z_* on Ra different.

Figure 14 shows the variation of surface roughness with ultrasonic amplitude *A_z_* and depth of cut *a_p_*. *a_p_* has a significant effect on both samples, and the roughness increases with the increase in *a_p_*. However, the effect of *a_p_* on sample B is more significant compared to sample A. This is mainly due to the increased cutting of the abrasive grains as the *a_p_*. increases. From the analysis of cutting forces described in Section 3.2, it can be seen that the cutting forces rise significantly with increasing *a_p_*, which leads to a decrease in the stability of the machining system and an increase in roughness.

In addition, the effect of *A_z_* on the roughness is also significant, and the roughness of the UAG surface was lower than that of the CG machining for both *A_z_* of 2 and 4 μm. This is mainly due to the lower cutting forces during UAG machining, which improves the stability of the machining system and reduces surface roughness. However, the roughness of sample A increased instead and even exceeded the roughness of CG machining when the *A_z_* exceeded 6 μm. The possible reason for this is that excessive ultrasonic vibration causes the shedding of particles, which results in abrasive scratches on the surface.

From the above analysis, it is clear that the roughness of sample A was lower than that of sample B under the same machining parameters, both in CG and UAG machining modes. This indicates that the surface roughness of the material tends to decrease as the volume fraction of the material increases. In addition, the Ra obtained with UAG machining was smaller than that obtained with CG machining for the same samples and with the same machining parameters.

### 3.4. Discussion

As shown in Figure 2, during the UAG machining process, a single diamond abrasive grain feeds the motion in the X direction and rotates at a high speed in the XY plane while vibrating at an ultrasonic frequency of 20 kHz along the Z direction. The trajectory of the abrasive grain in the X, Y, and Z directions can be described by the following equations:

(2) $$\left\{\begin{array}{l} X\left(t\right)=R\cos\left(\omega t\right)+v_{w}t\\ Y\left(t\right)=R\sin\left(\omega t\right)\\ Z\left(t\right)=A_{z}\sin\left(2\pi ft\right) \end{array}\right.$$

where *R* is the radius of the grinding tool; *f* is the ultrasonic vibration frequency; *t* is the machining time; *ω* is the angular velocity of tool rotation.

**Figure 15:**
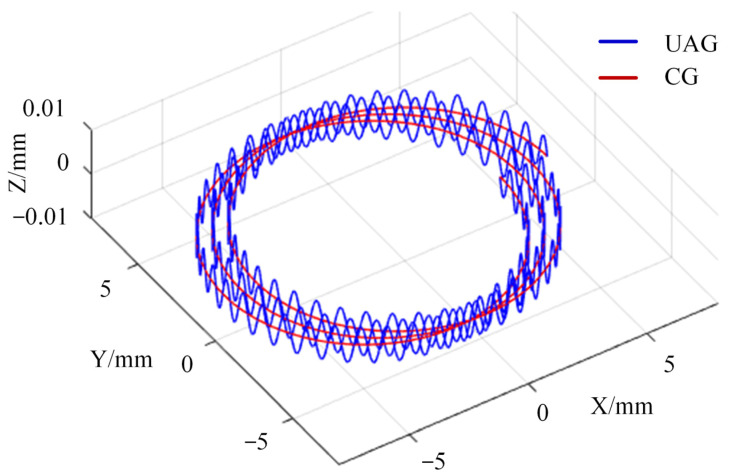
Simulation of abrasive grain trajectory.

The trajectory of a single abrasive grain simulated according to Equation (1) is shown in Figure 15. Unlike the planar motion trajectory of the abrasive grain under CG machining, the motion trajectory of UAG machining is three-dimensional. The length of the motion trajectory of the abrasive grain on the side of the grinding tool in the Z-direction is increased by the ultrasonic vibration, thus improving the material removal efficiency in the X-direction. The impact of the abrasive particles on the end face in the Z-direction creates a “hammering” effect, which also helps to remove SiCp particles. In addition, the intermittent cutting caused by ultrasonic vibration reduces the cutting force during the machining process, thus improving the stability of the machining system.

**Figure 16:**
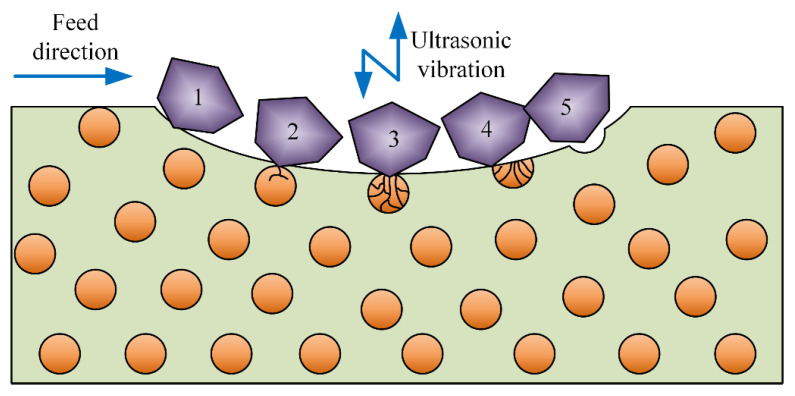
Material removal mechanism in 5 different cases.

Yin et al. pointed out that, during single-grain grinding, plastic removal of the aluminum matrix is accompanied by crack initiation, extension, and brittle fracture of SiCp particles [29]. Based on the SEM observations above, the removal mechanism between the aluminum matrix and SiCp particles is different. The aluminum matrix is mostly removed plastically, while the SiCp particles are mostly removed through brittle fracture. Based on the analysis of the publication [20], the UAG machining process can be categorized into five cases, as shown in Figure 16.

In case 1, the diamond grains are merely in contact with the aluminum matrix, and the material is removed plastically in this case. The ultrasonic action converts continuous cutting into intermittent cutting so that cutting fluid can easily enter the cutting area and chips can be easily discharged. In addition, the ultrasonic vibration enhances the aluminum matrix coating effect, so that some of the exposed SiCp particles can be covered by the aluminum matrix. As a result, the cutting force and surface roughness are reduced. Since the hardness of SiCp particles is significantly higher than that of the aluminum matrix, the cutting force is minimized in this case. As the volume fraction increases, the chances of case 1 will decrease, which leads to a subsequent increase in the cutting force.

According to the degree of contact between abrasive grains and SiCp particles, it can be categorized into cases 2–4. In case 2, the contact between abrasive grains and SiCp particles is quite slight, so that the SiCp particles are just beginning to crack but not completely broken. In case 3, the abrasive particles contact the SiCp particles deeply, so that the cracks in the SiCp particles continue to expand until they are completely broken. In case 4, the abrasive grains are in contact with completely crushed SiCp particles. In this case, only the broken particles need to be scraped away, so the cutting force is much smaller than in CG machining. The small and stable cutting force is very helpful in reducing the surface roughness. Case 5 is when most of the particles are exposed and only a small portion is embedded in the matrix. In this case, if the ultrasonic amplitude is high, the combined effect of the ultrasonic and rotary feed will cause the particles to be dislodged, resulting in craters [32]. Dislodged particles can scratch the surface or re-embed in the aluminum matrix. Since a higher volume fraction will lead to an increase in this case, surface roughness will increase subsequently.

## 4. Conclusions

Comparative machining tests were analyzed for two SiCp/Al materials with volume fractions of 45% and 60%, using both UAG and CG machining methods. The study mainly focuses on the machining effect of these two materials under different machining parameters, including machined surface morphology, chip force, and roughness. The following conclusions can be drawn:

(1) Compared to the 45% volume fraction SiCp/Al material, the processed surface of the 60% volume fraction SiCp/Al material had more surface defects and increased edge chipping. UAG machining with an amplitude of 4 μm or less reduces the depth and extent of hole defects as well as edge chipping defects. However, when the ultrasonic amplitude was increased to 8 μm, the pullout or shedding of SiCp particles increased, which resulted in more matrix scratch defects.
(2) UAG machining can effectively reduce the cutting force. UAG machining with an 8 μm ultrasonic amplitude decreased the cutting forces by 42.5% and 27.6%, respectively for the materials, compared to CG machining. In addition, the cutting force decreases with a decrease in feed rate, or depth of cut, and an increase in rotational speed. However, regardless of the machining parameters, the cutting forces of the 60% volume fraction SiCp/Al material were higher than those of the 45% volume fraction SiCp/Al material.
(3) For the same processing parameters, SiCp/Al with a volume fraction of 60% has a lower Ra than SiCp/Al with a volume fraction of 45%. Increasing the grinding speed or decreasing the feed rate will reduce the surface roughness. In addition, the Ra of the surface machined by UAG with a 4 μm ultrasonic amplitude was minimized. For UAG processing with ultrasonic amplitudes up to 4 μm, Ra decreases with increasing amplitude. However, for UAG machining with ultrasonic amplitudes exceeding 6 μm, Ra increases with increasing amplitude.
(4) Although SiCp/Al materials with volume fractions of 45% and 60% presented significant differences under UAG machining, the effect of UAG machining with ultrasonic amplitudes up to 4 μm was significant in reducing both cutting forces and roughness. This study provides a solution to enhance the processing quality of SiCp/Al materials. In the future, multi-objective optimization will be performed to obtain the optimal machining parameters for UAG machining of SiCp/Al materials with different body fractions.

## Figures and Tables

**Table 1:** Mechanical properties of samples A and B.

Sample No.	Volume Fraction	Tensile Strength	Yield Strength	Hardness	Elastic Modulus	Thermal Expansion Coefficient	Density
	(%)	(MPa)	(MPa)	(HBW)	(GPa)	(10^−6^/°C)	(g/cm^3^)
A	45	580	520	240	160	11.5	2.93
B	60	640	600	290	190	9.5	2.98

**Table 2:** Experimental conditions.

Groups	Sample	Processing Method	*v_s_* (krpm)	*v_w_* (mm/min)	*a_p_* (μm)	*A_z_* (μm)
1	A	CG	15	20, 60, 100, 140, 180, 220	10	0
2	A	UAG	15	20, 60, 100, 140, 180, 220	10	4
3	B	CG	15	20, 60, 100, 140, 180, 220	10	0
4	B	UAG	15	20, 60, 100, 140, 180, 220	10	4
5	A	CG	6, 9, 12, 15, 18, 21	120	10	0
6	A	UAG	6, 9, 12, 15, 18, 21	120	10	4
7	B	CG	6, 9, 12, 15, 18, 21	120	10	0
8	B	UAG	6, 9, 12, 15, 18, 21	120	10	4
9	A	CG/UAG	15	220	10	0, 2, 4, 6, 8
10	A	CG/UAG	15	220	20	0, 2, 4, 6, 8
11	B	CG/UAG	15	220	10	0, 2, 4, 6, 8
12	B	CG/UAG	15	220	20	0, 2, 4, 6, 8

## Data Availability

Data are contained within the article.

## Nomenclature

SiCp/Al	Silicon carbide particle-reinforced aluminum
SiCp	Silicon carbide particle
Sample A	Volume fractions of 45%
Sample B	Volume fractions of 60%
UAG	Ultrasonic-assisted grinding machining
CG	Conventional grinding
SEM	Scanning electron microscope
EDS	Energy dispersive spectroscope
*A_z_*	Ultrasonic amplitude
*a* *_p_*	Depth of cut
*v_w_*	Feed rate
*v_s_*	Tool speed
*R*	Radius of the grinding tool
*f*	Ultrasonic vibration frequency
*t*	Machining time
*ω*	Angular velocity of tool rotation

## References

[1] Liu P., Wang A.Q., Xie J.P., Hao S.M. (2015). Characterization and evaluation of interface in SiCp/2024 Al composite Pei. Trans. Nonferrous Met. Soc. China.

[2] Erdemir F., Canakci A., Varol T. (2015). Microstructural characterization and mechanical properties of functionally graded Al2024/SiC composites prepared by powder metallurgy techniques. Trans. Nonferrous Met. Soc. China.

[3] Cui Y., Wang L., Ren J. (2008). Multi-functional SiC/Al composites for aerospace applications. Chin. J. Aeronaut..

[4] Li Q., Yuan S., Gao X., Zhang Z., Chen B., Li Z., Batako A.D. (2023). Surface and subsurface formation mechanism of SiCp/Al composites under ultrasonic scratching. Ceram. Int..

[5] Yang W.-S., Chen G.-Q., Wu P., Hussain M., Song J.-B., Dong R.-H., Wu G.-H. (2017). Electrical Discharge Machining of Al2024-65 vol% SiC Composites. Acta Metall. Sin. Lett..

[6] Gao X., Li J., Xing Q., Zhang Q. (2022). Research on ultrasonic vibration–assisted electrical discharge machining SiCp/Al composite. Int. J. Adv. Manuf. Technol..

[7] Lu S., Gao H., Bao Y., Xu Q. (2019). A model for force prediction in grinding holes of SiCp/Al composites. Int. J. Mech. Sci..

[8] Dong X.W., Li Z.A., Bian L., Wang M., Jin Z.B., Da Li Q. (2023). Study on chatter suppression in ultrasonic-assisted grinding of thin-walled workpiece of SiCp/Al composites. Adv. Mech. Eng..

[9] Li Q., Yuan S., Batako A., Chen B., Gao X., Li Z., Amin M. (2024). Modeling for ultrasonic vibration-assisted helical grinding of SiC particle-reinforced Al-MMCs. Int. J. Adv. Manuf. Technol..

[10] Shi H., Yuan S., Zhang C., Chen B., Li Q., Li Z., Zhu G., Qian J. (2020). A cutting force prediction model for rotary ultrasonic side grinding of CFRP composites considering coexistence of brittleness and ductility. Int. J. Adv. Manuf. Technol..

[11] Cao Y., Ding W., Zhao B., Wen X., Li S., Wang J. (2022). Effect of intermittent cutting behavior on the ultrasonic vibration-assisted grinding performance of Inconel718 nickel-based superalloy. Precis. Eng. Int. Soc. Precis. Eng. Nanotechnol..

[12] Chen Y., Hu Z., Yu Y., Lai Z., Zhu J., Xu X., Peng Q. (2022). Processing and machining mechanism of ultrasonic vibration-assisted grinding on sapphire. Mater. Sci. Semicond. Process..

[13] Cao Y., Zhu Y., Ding W., Qiu Y., Wang L., Xu J. (2022). Vibration coupling effects and machining behavior of ultrasonic vibration plate device for creep-feed grinding of Inconel 718 nickel-based superalloy. Chin. J. Aeronaut..

[14] Hu C., Zhu Y. (2023). System Design and Mechanism Study of Ultrasonic-Assisted Electrochemical Grinding for Hard and Tough Materials. Processes.

[15] Ding K., Fu Y., Su H., Xu H., Cui F., Li Q. (2017). Experimental studies on matching performance of grinding and vibration parameters in ultrasonic assisted grinding of SiC ceramics. Int. J. Adv. Manuf. Technol..

[16] Denkena B., Friemuth T., Reichstein M. (2003). Potentials of different process kinematics in micro grinding. CIRP Ann. Manuf. Technol..

[17] Liu S., Chen T., Wu C. (2017). Rotary ultrasonic face grinding of carbon fiber reinforced plastic (CFRP): A study on cutting force model. Int. J. Adv. Manuf. Technol..

[18] Li Z., Yuan S., Song H., Batako A.D.L. (2018). A cutting force model based on kinematics analysis for C/SiC in rotary ultrasonic face machining. Int. J. Adv. Manuf. Technol..

[19] Jianhua Z., Hui L., Minglu Z., Yan Z., Liying W. (2017). Study on force modeling considering size effect in ultrasonic-assisted micro-end grinding of silica glass and Al_2_O_3_ ceramic. Int. J. Adv. Manuf. Technol..

[20] Zha H., Feng P., Zhang J., Yu D., Wu Z. (2018). Material removal mechanism in rotary ultrasonic machining of high-volume fraction SiCp/Al composites. Int. J. Adv. Manuf. Technol..

[21] Zhou M., Wang M., Dong G. (2016). Experimental Investigation on Rotary Ultrasonic Face Grinding of SiCp/Al Composites. Mater. Manuf. Process..

[22] Zhou M., Zheng W. (2016). A model for grinding forces prediction in ultrasonic vibration assisted grinding of SiCp/Al composites. Int. J. Adv. Manuf. Technol..

[23] Zheng W., Zhou M., Zhou L. (2017). Influence of process parameters on surface topography in ultrasonic vibration- assisted end grinding of SiCp/Al composites. Int. J. Adv. Manuf. Technol..

[24] Wang H., Zhang H., Zhou M. (2023). Study on surface defect formation mechanism in ultrasonic vibration-assisted grinding of SiCp/Al composites. Int. J. Adv. Manuf. Technol..

[25] Gu P., Zhu C., Sun Y., Wang Z., Tao Z., Shi Z. (2023). Surface roughness prediction of SiCp/Al composites in ultrasonic vibration-assisted grinding. J. Manuf. Process..

[26] Veličković S., Stojanović B., Babić M., Vencl A., Bobić I., Bognár G.V., Vučetić F. (2019). Parametric optimization of the aluminium nanocomposites wear rate. J. Braz. Soc. Mech. Sci. Eng..

[27] Velickovic S., Stojanović B., Djordjević Z., Miladinović S., Blagojević J. (2019). Effect of reinforcement on mechanical characteristics of A356 alloy nanocomposites. IOP Conf. Ser. Mater. Sci. Eng..

[28] Xiang D., Liu G., Peng P., Zhang Z. (2022). Micro-removal characteristics of SiCp/Al by ultrasonic vibration-assisted scratch. Mater. Manuf. Process..

[29] Yin G., Gong Y., Li Y., Song J., Zhou Y. (2019). Modeling and evaluation in grinding of SiCp/Al composites with single diamond grain. Int. J. Mech. Sci..

[30] Gao Q., Guo G., Wang Q. (2021). Study on micro-grinding mechanism and surface quality of high-volume fraction SiCp/Al composites. J. Mech. Sci. Technol..

[31] Zhou L., Wang Y., Ma Z.Y., Yu X.L. (2014). Finite element and experimental studies of the formation mechanism of edge defects during machining of SiCp/Al composites. Int. J. Mach. Tools Manuf..

[32] Zhang X., Yang L., Wang Y., Lin B., Dong Y., Shi C. (2020). Mechanism study on ultrasonic vibration assisted face grinding of Hard and brittle materials. J. Manuf. Process..

